# Similarities and Differences in the Effects of Toxic Concentrations of Cadmium and Chromium on the Structure and Functions of Thylakoid Membranes in *Chlorella variabilis*

**DOI:** 10.3389/fpls.2020.01006

**Published:** 2020-07-07

**Authors:** Ottó Zsiros, Gergely Nagy, Roland Patai, Katalin Solymosi, Urs Gasser, Tamás F. Polgár, Győző Garab, László Kovács, Zsolt Tibor Hörcsik

**Affiliations:** ^1^ Institute of Plant Biology, Biological Research Centre, Szeged, Hungary; ^2^ Laboratory for Neutron Scattering and Imaging, Paul Scherrer Institute, Villigen PSI, Villigen, Switzerland; ^3^ Institute for Solid State Physics and Optics, Wigner Research Centre for Physics, Budapest, Hungary; ^4^ Neutron Scattering Division, Oak Ridge National Laboratory, Oak Ridge, TN, United States; ^5^ Institute of Biophysics, Biological Research Centre, Szeged, Hungary; ^6^ Department of Plant Anatomy, ELTE Eötvös Loránd University, Budapest, Hungary; ^7^ Department of Physics, Faculty of Science, University of Ostrava, Ostrava, Czechia; ^8^ Department of Biology Nyíregyháza, Institute of Environmental Sciences, University of Nyíregyháza, Nyíregyháza, Hungary

**Keywords:** cadmium, chromium, circular dichroism, electron microscopy, green alga, P700, photosystem II, small-angle neutron scattering

## Abstract

Trace metal contaminations in natural waters, wetlands, and wastewaters pose serious threats to aquatic ecosystems—mainly *via* targeting microalgae. In this work, we investigated the effects of toxic amounts of chromium and cadmium ions on the structure and function of the photosynthetic machinery of *Chlorella variabilis* cells. To halt the propagation of cells, we used high concentrations of Cd and Cr, 50–50 mg L^−1^, in the forms of CdCl_2_ x 2.5 H_2_O and K_2_Cr_2_O_7_, respectively. Both treatments led to similar, about 50% gradual diminishment of the chlorophyll contents of the cells in 48 h, which was, however, accompanied by a small (~10%) but statistically significant enrichment (Cd) and loss (Cr) of ß-carotene. Both Cd and Cr inhibited the activity of photosystem II (PSII)—but with more severe inhibitions with Cr. On the contrary, the PsbA (D1) protein of PSII and the PsbO protein of the oxygen-evolving complex were retained more in Cr-treated cells than in the presence of Cd. These data and the higher susceptibility of P700 redox transients in Cr-treated cells suggest that, unlike with Cd, PSII is not the main target in the photochemical apparatus. These differences at the level of photochemistry also brought about dissimilarities at higher levels of the structural complexity of the photosynthetic apparatus. Circular dichroism (CD) spectroscopy measurements revealed moderate perturbations in the macro-organization of the protein complexes—with more pronounced decline in Cd-treated cells than in the cells with Cr. Also, as reflected by transmission electron microscopy and small-angle neutron scattering, the thylakoid membranes suffered shrinking and were largely fragmented in Cd-treated cells, whereas no changes could be discerned with Cr. The preservation of integrity of membranes in Cr-treated cells was most probably aided by high proportion of the de-epoxidized xanthophylls, which were absent with Cd. It can thus be concluded that beside strong similarities of the toxic effects of Cr and Cd, the response of the photosynthetic machinery of *C. variabilis* to these two trace metal ions substantially differ from each other—strongly suggesting different inhibitory and protective mechanisms following the primary toxic events.

## Introduction

Aquatic ecosystems may extensively be contaminated with heavy metals arising from domestic and industrial activities. This may, in turn, have devastating effects on these ecological units (Baby et al., 2010; Yılmaz et al., 2017). In general, toxic effects of trace metal ions are related to the production of reactive oxygen species (ROS), which, in turn, induces adverse effects, a variety of reactions depending on the organism, the chemical nature and concentration of the heavy metals, and some additional factors (Pinto et al., 2003; Mota et al., 2015). Among the trace metal ions, subjects of the present study, cadmium and chromium are both potentially highly toxic ions, which retard the growth of green algae and inhibit their photosynthetic functions (Kumar et al., 2014; Machado and Soares, 2015; Mota et al., 2015).

Cadmium is a rare element in the earth’s crust. Its elevated levels arise mainly from mining, non-ferrous metal production, disposal of NiCd rechargeable batteries and electronic products, phosphate fertilizers, and manure. Cd is one of most dangerous of heavy metals, mainly due to its relatively long biological half-time. Plants and phytoplankton accumulate and store this element for long time, which can thus be transferred to higher trophic levels of the food chain (Conway, 1978). Its half-time in human body can be as long as 25–50 years (Wagner, 1993).

In general, Cd^2+^ induces oxidative stress, manifested, among others, in increased lipid peroxidation and depletion of ascorbate and glutathione stores (Maret and Moulis, 2013). The toxic effect of Cd^2+^ on photosynthesis may involve several different mechanisms (Vanassche and Clijsters, 1990; Grzyb et al., 2004; Faller et al., 2005). In the cyanobacterium *Synechocystis* PCC 6803 toxic amounts of Cd ions led to the degradation of the D1 protein of PSII and the gradual loss of all photochemical activity of the thylakoid membranes, which, however, has been shown to be triggered by inhibitory effects in the dark reactions of photosynthesis (Tóth et al., 2012). In the cyanobacterium *Nostoc entophytum*, cadmium exposure of cells appeared to trigger adverse effects, series of events, including destruction of some pigment-protein complexes, changes in catalase activity and lipid peroxidation and membrane damage, as well as protective mechanisms, such as the induction of chaperon proteins (Alidoust et al., 2019). In the freshwater green alga *Micrasterias denticulata*, inhibition of PSII activity and the damage in chloroplast ultrastructure has been shown to arise from a disturbance of Ca homeostasis probably *via* displacement of Ca by Cd (Andosch et al., 2012; Volland et al., 2014). Toxic concentrations of Cd in *Chlorella vulgaris* led to decreased chlorophyll and protein contents, evidently due to increased levels of H_2_O_2_ and O_2_^·−^—but antioxidant protective mechanisms were also activated *via* SOD, catalase and peroxidase, and glutathione reductase enzymes (Cheng et al., 2016). In *Chlamydomonas reinhardtii* exposed to 600 μM CdCl_2_, which slowed down the cell division by about 30%, Cd ions accumulated relatively rapidly in the chloroplasts—explaining its ~50% inhibitory effect on PSII in 24 h; at the same time no noticeable damage in the ultrastructure was seen (Samadani et al., 2018). Possible targets for direct effects of Cd were identified in barley thylakoids as the Mn cluster, plastocyanin, carbonic anhydrase, and the FtsH protease (Lysenko et al., 2019).

Chromium is the seventh most abundant metal in the earth’s crust. It is nonessential for plant and algae and its toxicity depends on its valence state and concentrations. Unlike the metallic form, Cr(0), and the Cr(III) ion, which are not toxic, Cr(VI) is highly toxic. Hexavalent Cr(VI) in the environment and in surface waters is almost totally derived from human activities, electroplating, leather tanning, and textile industries (Yu et al., 2011).

The toxicity of Cr(VI) is manifested in its capability of inducing oxidative stress, due to the formation of ROS upon the conversion of the hexavalent form to Cr(III) in the organisms. This, in principle, accounts for its phytotoxic effects, inhibition of growth, and degradation of photosynthetic pigments (Panda and Choudhury, 2005). However, details of the mechanisms of the reduction of Cr(VI) to Cr(III) on the organelle level, and in algal chloroplasts, in particular, are still unclear (Chen et al., 2016). Further, the toxicity of chromium can be recognized at several different levels of the structural and functional complexity of the photosynthetic machinery. The enhanced oxidative stress and lipid peroxidation in *Chlorella pyrenoidosa* treated with up to 40 mg L^−1^ (0.7 mM) Cr led to the photodestruction of D1 protein of PSII. High concentration (1 mM) of Cr, administered to the green alga *Micrasterias denticulata*, revealed the rapid generation of ROS demonstrating the efficient uptake of Cr by the cells; which in turn, activated some antioxidant protection mechanisms and also led to deteriorations in the chloroplast ultrastructure (Volland et al., 2012). (Short-term toxic effects, used in this study were similar to the long-term effects induced by much lower Cr concentrations in several weeks.) Nevertheless, complexity of the toxic effects in green algae is shown by the several independent studies and observation of adverse effects and different factors determining the apparent nature and magnitude of damage. The exposure of wild type and mutant *Chlamydomonas reinhardtii* to Cr led to different magnitudes of PSII photoinhibition, depending on the functional activity of the xanthophyll cycle (XC) (Ait Ali et al., 2008)—suggesting the active protective roles of the non-photochemical quenching (Demmig-Adams et al., 2014) and of the antioxidant activity of zeaxanthin (Zx) (Havaux et al., 2007). It is equally interesting that the availability of inorganic phosphate largely alleviated the Cr-toxicity induced damage to thylakoid membranes in *Chlorella vulgaris* (Qian et al., 2013).

As outlined above, the most common feature, and basis of the toxic effects of both Cd and Cr on the photosynthetic machinery in algae is that these trace metal ions induce oxidative stress. It is also clear, as revealed by several studies that—possibly as consequences of the primary effect of ROS production—there are adverse inhibitory effects, damages, and protective mechanisms. However, these were uncovered using different organisms and experimental conditions—and thus no firm conclusion can be reached concerning the putative similarities and possible differences in the mechanisms of toxicity and defense in the presence of Cd and Cr. A step in this direction, in the present study we carried out systematic experiments on the effects of these trace metal ions on the structure and functions of the photosynthetic machinery in *Chlorella variabilis*—and show that beside some basic similar features there are important differences between the Cd- and Cr-induced stress responses at the primary level of photochemical reactions, the pigment compositions, and the levels of macroorganization of the protein complexes and the thylakoid membranes.

## Materials and Methods

### Alga Cultures and Conditions; Determination of Cell Numbers

*Chlorella variabilis* 211-6 cultures were grown in Tris-acetate-phosphate (TAP) medium at a light intensity of 100 μmol photons m^−2^ s^−1^ at 24–25°C. One hundred ml Erlenmeyer ﬂasks containing 50 ml TAP medium were shaken at 120 rpm and the cultures were grown for 4 d before use. Cells were counted with Dual Fluorescence Cell Counter (Luna™ *fl*, Logos).

### Cadmium and Chromium Treatments

Four-day old algal cultures were treated with CdCl_2_ x 2.5 H_2_O (Sigma) or K_2_Cr_2_O_7_ (Sigma). The trace metal ion content was 50 mg L^−1^—corresponding to 446 and 942 µM Cd^2+^ and Cr^2+^, respectively. These amounts inhibited about equally the cell growth.

### Pigment Determination

For pigment determination 2 ml aliquots of alga cultures were spun down and the cells were resuspended in 2 ml 90% acetone. Pigments were extracted for 30 min with continuous shaking at 1,000 rpm at 20°C in the dark. The extract was centrifuged at 11,500 *g* for 10 min at 4°C, and the supernatant was collected and passed through a PTFE 0.2 μm pore-size syringe filter.

Quantification of carotenoid contents was performed by HPLC, using a Shimadzu Prominence HPLC system (Shimadzu, Kyoto, Japan) consisting of an LC-20AD pumps, a DGU-20A degasser, a SIL-20AC automatic sample injector, CTO-20AC column thermostat, and a Nexera X2 SPD-M30A photodiode-array detector. Chromatographic separations were carried out on a Phenomenex Synergi 4 µm Hydro-RP 80Å, 250 x 4.6 mm column. Twenty microliter aliquots of acetonic extract were injected to the column and the pigments were eluted by a linear gradient from solvent A (acetonitrile, water, triethylamine, in a ratio of 9:1:0.01) to solvent B (ethylacetate) followed by 15 min re-equilibration in solvent A. The gradient from solvent A to solvent B was run from 0 to 25 min at a flow rate of 1 ml min^−1^. The column temperature was set to 25°C. Eluates were monitored in a wavelength range of 260–750 nm. Pigments were identified according to their retention time and absorption spectrum and quantified by integrated chromatographic peak area recorded at the wavelength of maximum absorbance for each kind of pigments using the corresponding molar decadic absorption coefficient (Jeffrey et al., 2005).

### Fast Chlorophyll-A Fluorescence (OJIP)

Fast Chl-a ﬂuorescence measurements were carried out at room temperature with a Handy-PEA instrument (Hansatech Instruments Ltd, UK). *C. variabilis* cells were dark acclimated for 15 min and then 3 ml of cell suspension (10 μg Chl ml^−1^) was ﬁltered onto a Whatman glass microﬁber ﬁlter (GF/B). The sample was illuminated with continuous red light (3,500 μmol photons m^−2^ s^−1^) for 3 s. The ﬁrst reliably measured point of the ﬂuorescence transient is at 20 μs, which was taken as F_o_.

### Oxidation–Reduction Kinetics of P700

The light-induced redox changes of P700 were monitored by measuring the absorbance changes at 820 nm with a Dual PAM-100 chlorophyll fluorometer (Heinz Walz). The sample (equivalent to 20 µg Chl) was filtered onto a filter paper disk and the cells were dark acclimated for 3 min before the measurements. In order to determine the amount of total photo-oxidizable P700, 10 s far-red pre-illumination followed by a 200 ms long pulse with 1,000 µmol photons m^−2^ s^−1^ maximum photon flux density was used.

### Western Blot Analysis

At each time point, 1 ml of culture were collected, spun down for removal of the supernatant and frozen in liquid N_2_. Melted samples were resuspended in 250 µl 2x Laemmli buffer. The same volume of 425–600 µm glass beads (Sigma) were added and the cells were homogenized at 8,000 RPM for 30 s 6 times with 30 s intervals in a Precellys Evolution homogeniser (Bertin instruments). The homogenates were incubated at 65°C for 5 min and centrifuged for removal of cell debris. Proteins separated by 15% Tris-Glycine-SDS-PAGE (Hoefer SE250 Gel System) were transferred to a polyvinylidene difluoride membrane (Hybond P) with a semidry blotting system (Cleaver Scientific Ltd) using methanol-containing buffer (25 mM TRIS, 192 mM glycine, 20% methanol). The membranes were blocked using 5% milk powder in TBST buffer (10 mM TRIS pH 8.0, 150 mM NaCl, 0.1% Tween 20) for 1 h and incubated with primary antibodies in TBST buffer for 1 h. Specific polyclonal antibodies (produced in rabbits) against PsbA and PsbO were purchased from Agrisera AB. As secondary antibody, a goat anti-rabbit IgG horseradish peroxidase conjugate (Millipore) at a 1:5,000 dilution in TBST buffer was applied for 1 h. Immunochemical detection was carried out with the ECL Prime System (GE Healthcare), according to the instructions of the manufacturer.

### Circular Dichroism Spectroscopy

CD spectra of intact *C. variabilis* cells were recorded at room temperature using a Jasco J-815 spectropolarimeter. The spectra were recorded between 400 and 800 nm with bandwidth of 2 nm and data pitch of 1.0 nm. The scan speed was set to 100 nm/min and the integration time was 1 s. The CD spectra were normalized to the red-most absorbance maxima, at around 690 nm, with a reference wavelength at 750 nm.

### Specimen Preparation for Electron Microscopy

*C. variabilis* cells were fixed in Karnovsky solution (Karnovsky, 1965) containing 2% paraformaldehyde (Sigma; St. Louis, MO, USA) and 2.5% glutaraldehyde (Polysciences; Warrington PA, USA) in phosphate buffer for overnight at 4°C. After fixation, samples were rinsed in distilled water (pH 7.4) for 10 min followed by a 2% OsO_4_ solution (in distilled water, pH 7.4) for 60 min (Millonig, 1961). After osmification, samples were briefly rinsed in distilled water for 10 min, then dehydrated through a graded series of ethanol (from 50% to 100%; Molar; Halasztelek, Hungary) for 10 min in each concentration and proceeded through propylene oxide. Dehydrated samples were embedded in an epoxy-based plastic (Durcupan ACM; Sigma) then polymerized at 56°C for 48 h. Fifty nm ultrathin sections were cut from the plastic blocks on an Ultracut UCT ultramicrotome (Leica; Wetzlar, Germany) and placed on single-hole formvar-coated copper grids (Electron Microscopy Sciences; Hatfield, PA, USA). Ultrathin sections were contrasted with 2% uranyl acetate (Electron Microscopy Sciences) in 50% ethanol (Molar) and 2% lead citrate (Electron Microscopy Sciences) in distilled water (Reynolds, 1963; Hayat, 1970).

### Electron Microscopic Morphometry of Thylakoid Membranes

Samples were evaluated under a JEM-1400Flash transmission electron microscope (JEOL; Tokyo, Japan) to identify the morphological characteristics of the thylakoid membranes. The specimens were systematically screened at 5,000× magnification to localize the cells on the grid. Afterward, thylakoid membranes were recorded at 30,000–100,000× magnification with a 16 MP Matataki Flash scientific complementary metal–oxide–semiconductor (sCMOS) camera (JEOL). Quantitative analysis of the thylakoid-membrane repeat distances was conducted *in situ*, using the built-in tools of the transmission electron microscope. Data of these measurements represented as mean ± standard error of the mean.

### Small-Angle Neutron Scattering

The experiments were performed on the small-angle neutron scattering (SANS) II instrument of the Paul Scherrer Institute (PSI, Villigen, Switzerland). The applied settings for the measurements of the samples were: SD=1.22 m; collimation, 2 m; λ=5.52 Å; and SD=4 m; collimation, 4 m; λ=5.52 Å for the high-Q and low-Q, regions, respectively. (SD, sample-to-detector distance; Q, momentum transfer). As a subtractable sample background, we measured the D_2_O-based TAP buffer, which was used as a suspension buffer for the algal cells during the measurements. For detector efficiency calibration the measurements were performed on Cd plate, cuvette and H_2_O in a quartz cuvette with 1 mm path length and 1 cm width, with instrument setting of SD, 1.22 m; collimation, 2 m; and λ, 5.52 Å. For data treatment the “Graphical Reduction and Analysis SANS Program” package (GRASP) (developed by C. Dewhurst, ILL) was used. The samples were measured in a sample holder with permanent magnets providing a ~ 0.4 T magnetic field in the horizontal direction perpendicular to the neutron beam. The orientation of the samples was clearly visible on the 2D scattering images. Consequently, during the data treatment, two sectors with 90° opening angle were used for radial averaging of the 2D scattering images. The 1D curves were fitted with the sum of four Gaussians and a power function, similar to the method described earlier (see Nagy et al., 2013).

### Statistics

All statistical analyses were carried out using OriginPro 8 program. All measurements were performed by at least 3 independent biological replicates. The exact number of replicates are indicated in the figure legends. Data are expressed as mean ± SE. Significance levels were tested by one-way ANOVA at p<0.05. Multiple comparison of the means were performed by Bonferroni post-hoc test.

## Results and Discussion

### Effects of Cd and Cr on Growth and Pigment Contents

**Figure 1:**
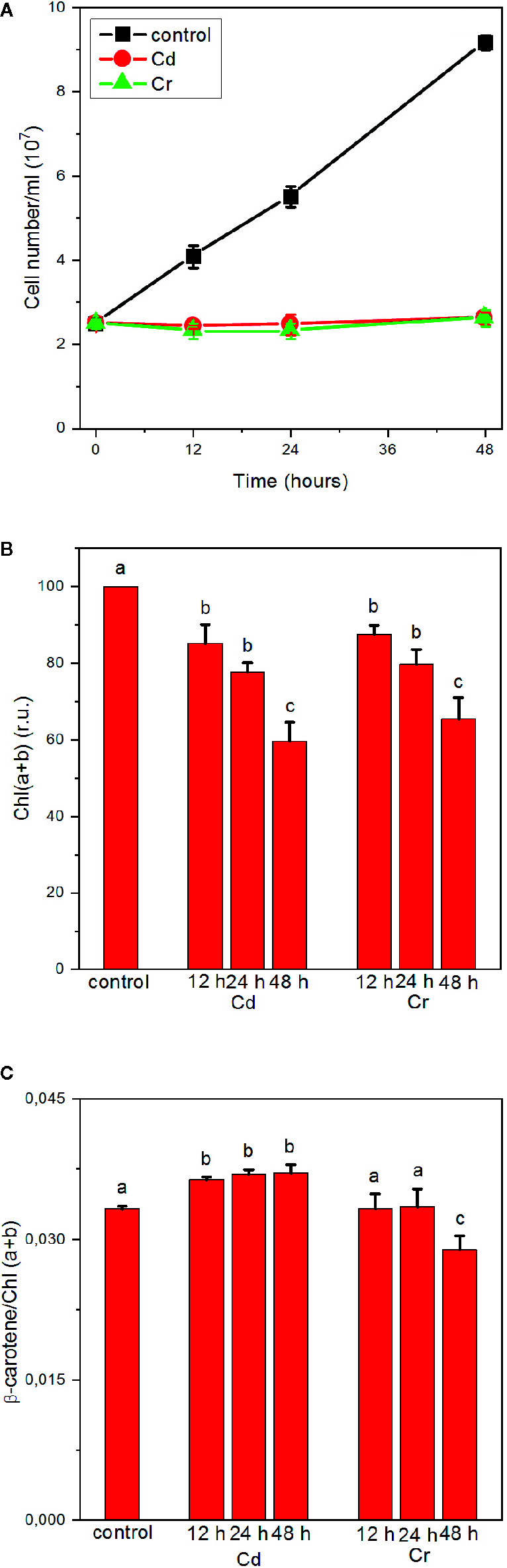
Effects of cadmium and chromium ions, 50 mg L^−1^ each, on the growth (cell number) **(A)**, Chl (a+b) contents **(B)** and ß-carotene/Chl (a+b) ratio of *C. variabilis* cells; mean values ± SE from five independent experiments. Different letters in panels **(B**, **C)** indicate statistically significant differences (ANOVA with Bonferroni post-hoc test, P<0.05).

As shown in **Figure 1A**, high concentrations, 50–50 mg L^−1^, of Cd and Cr blocks the division of cells, i.e. halts the growth of the culture, but without leading to noticeable decrease in the number of cells. Using high concentrations of trace metal ions, considerably higher than the concentrations found in contaminated waters (up to 100 µg/L for Cd and 22 µg/L for Cr) (Kabata-Pendias and Pendias, 2001; Frey et al., 2004; Oliveira, 2012; Idrees et al., 2018), offers the possibility of an easier monitoring of the rapidly developing effects of toxicity (Volland et al., 2012). The treated samples suffer gradual bleaching, an approx. 40% decrease in the Chl(a+b) content of the cells in 48 h both in the presence of Cd and Cr (**Figure 1B**). At the same time, only small, statistically insignificant variations between 3.5 and 3.6 were seen in the Chl a/b ratio, except after 48 h Cr-treatment, in which it decreased to 3.28 (data not shown). Small but statistically significant changes were observed in the ß-carotene/Chl(a+b) ratios, an ~10% increase in all Cd-treated samples, and decrease of about the same magnitude after 48 h in the Cr-treated cells, suggesting some enrichment and depletion, respectively, of the core complexes relative to the light-harvesting antennae. It is also interesting to note that albeit the changes are small, the variations in the presence of Cd and Cr are of opposite direction.

### Distinct Inhibitory Effects of Cd and Cr on the Photochemical Apparatus

**Figure 2:**
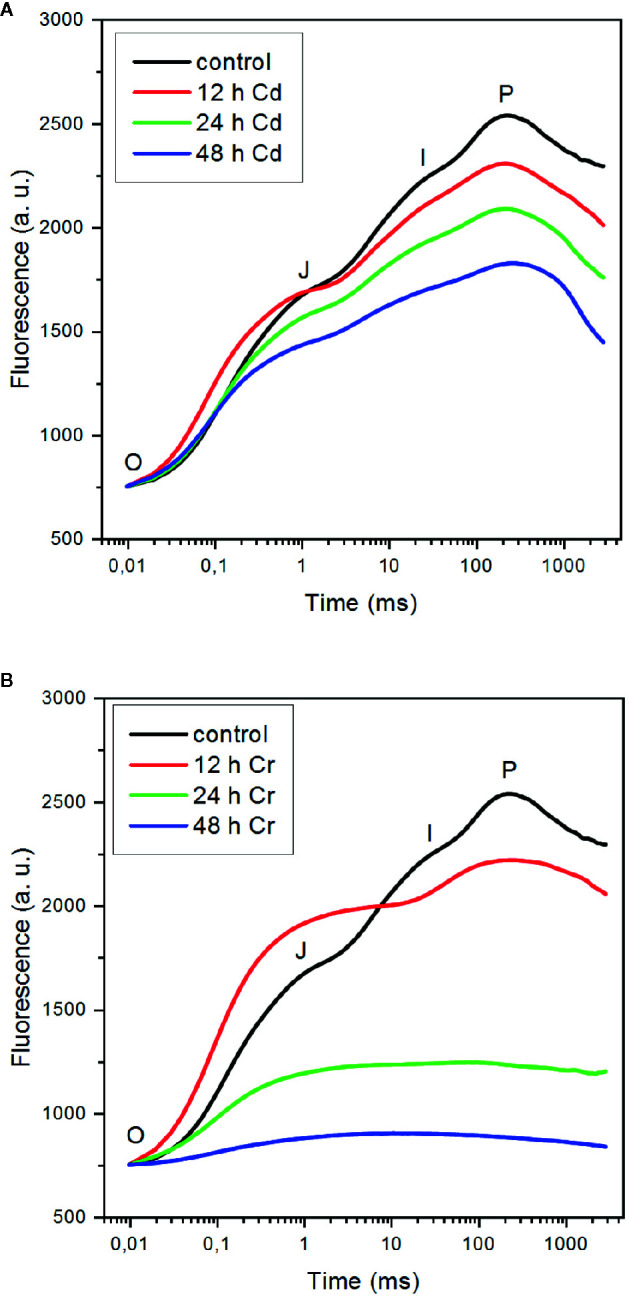
Effect of cadmium **(A)** and chromium **(B)** ions on the fast Chl-a fluorescence (OJIP) transients of *C. variabilis* cells 12, 24, and 48 h after the trace metal treatments of 50 mg L^−1^ ion contents, compared to the untreated cells. The kinetic traces represent the averages from five independent experiments on different batches. The traces are normalized to Fo.

As shown in **Figure 2**, both Cd (A) and Cr (B) strongly affected the fast Chl a (OJIP) fluorescence transient. The polyphasic rise of the variable fluorescence (F_v_) is a sensitive indicator of the photosynthetic electron transport processes (Govindjee, 2004; Strasser et al., 2004; Lazar, 2006; Tóth et al., 2007). The first (OJ) phase, which is conventionally referred to as the photochemical phase, is associated with the reduction of the primary quinone acceptor Q_A_ of PSII; the JI step, occurring between about 3 and 30 ms, is related to the reduction of the PQ pool, and the rise from level I to the P maximum is correlated with the reduction of PSI acceptor side.

It can be seen that in the presence of Cd, the overall shape of the OJIP transient is only moderately affected (**Figure 2A**). After 12 h the relative intensity of the OJ phase somewhat increased compared to the other phases, but afterward the magnitude of the variable fluorescence was gradually suppressed with kinetic features similar to the control. This suggests that (i) inhibition of the PSII charge separation, i.e. the reduction of Q_A_, is not the primary target of Cd—in good accordance with our earlier findings on cyanobacterial cells (Tóth et al., 2012); (ii) in later phases (24–48 h) of Cd-toxicity, the OJIP curves can be interpreted as an overall suppression of the photochemical activity of the membranes, in which the remaining active PSII centers were feeding electrons into the remaining whole chain electron transport system; and (iii) the inactive centers contributed to the fluorescence, gradually elevating the F_o_ level from 29% (t=0) to 37% (12 h), 41% (t=24 h), and 56% (t=48 h) relative to Fm(≡P) (data not shown). Although it is clear that the primary site of action of Cd is not PSII, the significance of PSII inhibitory site is marked by the observation that in Cd-resistant *Chlamydomonas reinhradtii* the inhibitory effects detected by OJIP transients were largely eliminated (Yu et al., 2018). The genes involved in photosynthesis, glutathione metabolism, and calcium transport were related to Cd tolerance.

In the presence of Cr, the OJIP curves exhibited different features compared to the control and the Cd-treated samples (**Figure 2B**): (i) after 12 h of the treatment, the fluorescence rise was dominated by the OJ phase and the contribution of the JI and IP phases were unusually small—a rise kinetics similar to that obtained on 50 μM Cr-treated pea after 96 h (Todorenko et al., 2020), which indicated that the earliest effect on the photosynthetic electron transport chain was in the intersystem electron transport; (ii) similar to the OJIP transients on Cr-treated pea, we also observed a pronounced rise of the F_o_ level to 44% at 12 h (data not shown), which was attributed to the partial reduction of Q_A_ (Todorenko et al., 2020); (iii) in later phases of the toxic effects, the JI and IP phase were absent and the F_o_ level, which probably also contained contributions from inactive PSII complexes, rose further, to 76% (t=24 h) and 93% (t=48 h), virtually eliminating the F_v_. It is interesting to note that the fluorescence rise observed in the sample treated with high concentration (50 mg L^−1^, corresponding to 962 μM) Cr also closely resembles the transient of *M. denticulata* treated with 10 μM Cr(VI) for 3 weeks (Volland et al., 2012). These authors also observed that Cr toxicity doubled the rate of respiration while reduced the net oxygen evolution to 10% of the original value.

**Figure 3:**
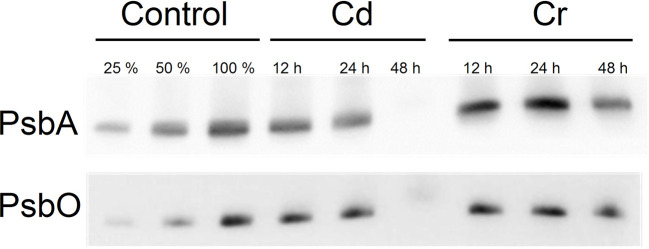
Western blot analysis for the PsbA and PsbO proteins of Control, Cd and Cr treated *Chlorella variabilis* cultures. The first two lanes (25%, 50% of control samples) are included for approximate quantification of the proteins.

In reasonable agreement with the data above, we found that the D1 protein of PSII and the PsbO protein of the oxygen-evolving complex (OEC) degraded considerably faster in the presence of Cd than with Cr. In the presence of Cr large fractions of these proteins were retained even 48 h after the treatment, whereas with Cd, only traces of these key proteins of PSII photochemistry were seen (**Figure 3**). These findings support the notion that Cd and Cr exhibit distinct mechanisms of the complex series of toxic effects on the light reactions of photosynthesis.

**Figure 4:**
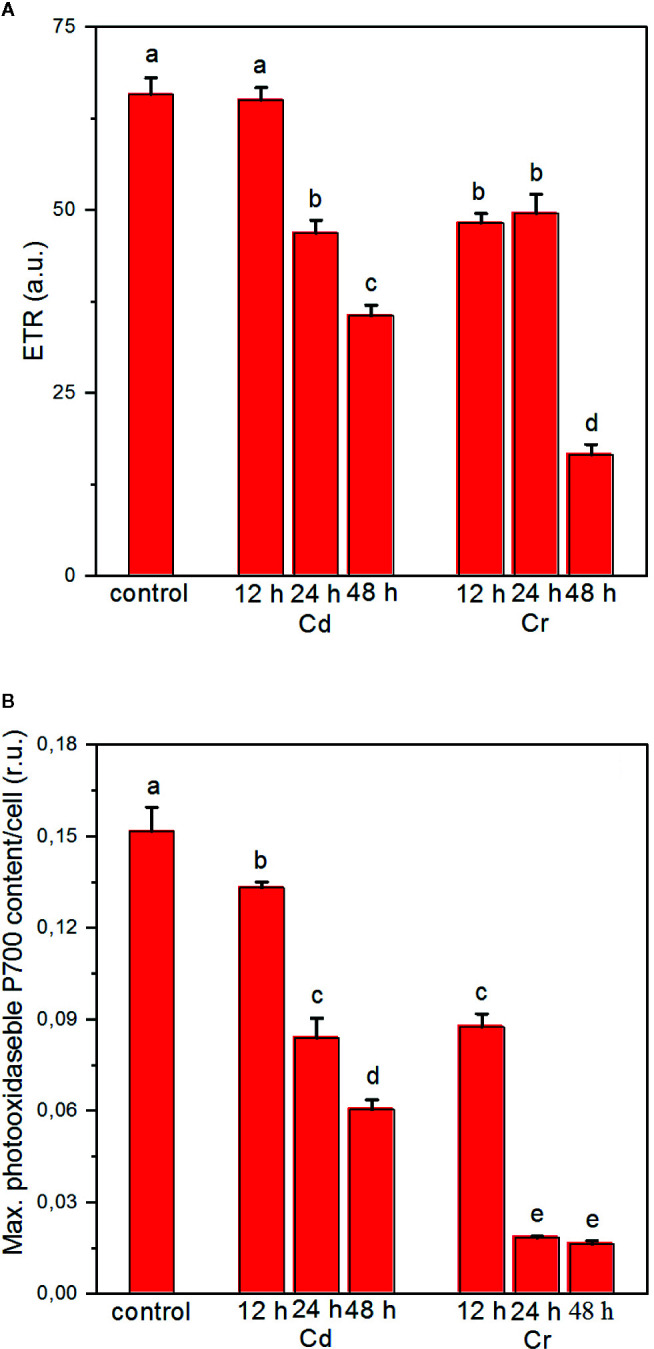
Effects of cadmium and chromium ions on the photosynthetic electron transport rate (ETR) **(A)** and the light-induced redox turnover of the PSI reaction center P700 **(B)** of *C. variabilis* cells at 12, 24, and 48 h after the trace metal treatment of 50 mg L^−1^ ion contents, compared to the untreated (control) cells. Mean values ± SE from five independent experiments on different batches. Different letters indicate statistically significant differences (ANOVA with Bonferroni post-hoc test, P<0.05).

In good agreement with the conclusion that upon Cr treatment of the cells, the strongest effects on the photochemical apparatus are found around the cytochrome b/f complex and PSI (Todorenko et al., 2020), we also found that the linear electron transport rate (ETR) and the photooxidizable concentration of P700 are severely inhibited (**Figures 4A, B**, respectively). These inhibitory effects on ETR and on the activity of PSI were less pronounced with Cd. Indeed, in cyanobacteria in the presence of Cd, light-dependent ROS accumulation has been shown to lead to the degradation of the D1 protein and to block its repair mechanism, and to exert only minor influence on the redox transients of P700 (Tóth et al., 2012; Du et al., 2019). In *Chlorella pyrenoidosa* PSII exhibited higher sensitivity to Cd exposure than PSI and it is suggested that the PSI-dependent cyclic electron transport can alleviate the harmful effect of Cd on PSI (Wang et al., 2013). Direct effect of Cd on the donor side of PSII has also been demonstrated in *Chlamydomonas reinhardtii* (Faller et al., 2005); photoactivation of PSII is inhibited upon Cd treatment at a low concentration. The inactivation of PSII water splitting capability increases the production of ROS *via* donor-side induced photoinhibition (Vass, 2012). The accumulation of ROS results in the degradation of PSII protein subunits, mainly the RC, and also the bleaching of chlorophylls. Cadmium-induced oxidative stress causes not only protein oxidation but also lipid peroxidation. Degradation and chemical modification of membrane lipids (Shah et al., 2001) can interrupt the thylakoid membrane integrity.

### Perturbation of the Macro-Organization of the Protein Complexes by Cd and Cr

Consequences of the toxic effects of Cd and Cr on the pigment contents and protein complexes led to well discernible alterations in the macro-organization of the protein complexes in the thylakoid membranes, as revealed by CD spectroscopy. CD spectroscopy is a noninvasive technique, which provides information on the organization of the pigment systems in hierarchically organized molecular assemblies (Garab and van Amerongen, 2009). The giant psi-type CD signals reflect the long-range chiral order of the chromophores in the pigment–protein complexes; it is superimposed onto their individual excitonic CD signals given rise by short-range pigment-pigment interactions. The psi-type CD is sensitive to both the lateral order of the complexes and the stacking of membranes and also depends on the size and composition of the chiral macrodomains which, in higher plants and green algae are formed by the PSII–LHCII supercomplexes (Garab et al., 1991; Goss and Garab, 2001; Kovács et al., 2006; Nagy et al., 2014). The psi-type CD bands of thylakoids are found at around (+)686–690 nm, (−)672–676 nm and at around (+)505–510 nm; these bands have been shown to possess distinct sensitivities to variations in the osmotic and ionic strengths and respond in different manner to the stacking interactions of membranes (Lambrev and Akhtar, 2019). The main positive psi-type bands in the red have been shown to depend largely on the LHCII content of the membranes, while the (+)blue band appears only in the presence of PSII–LHCII supercomplexes and does not depend on the xanthophyll composition of the membranes (Tóth et al., 2016).

**Figure 5:**
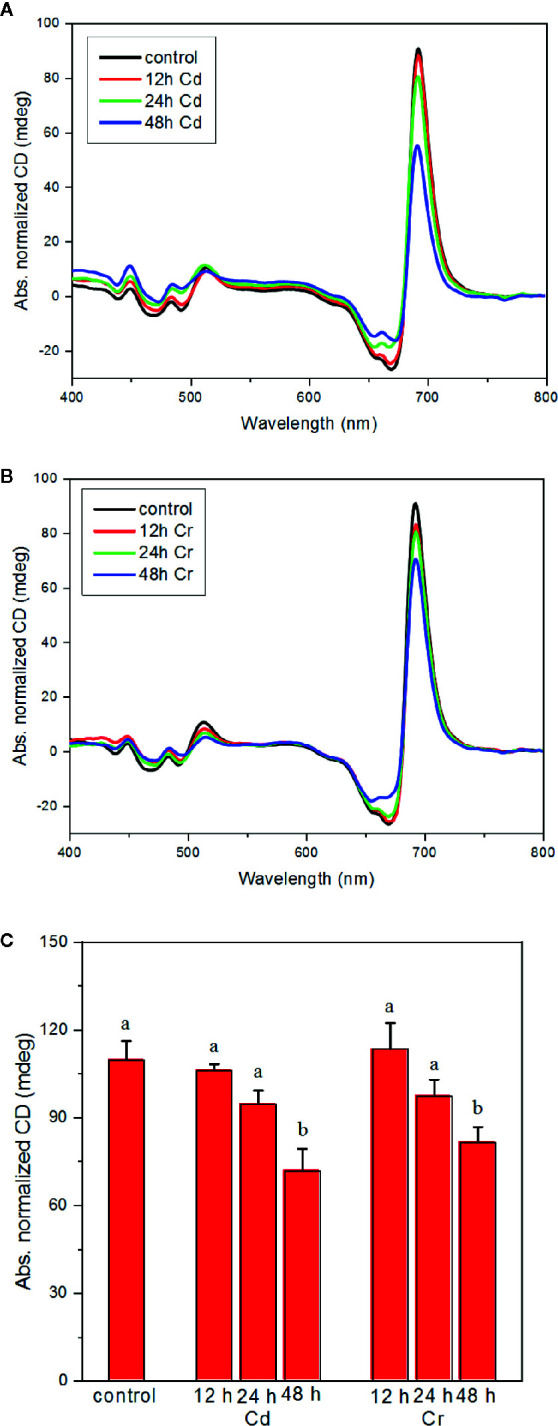
Effect of cadmium **(A)** and chromium **(B)** ions on the CD spectra and on the magnitude of the psi-type bands **(C)** of *C. variabilis* cells 12, 24, and 48 h after the trace metal treatment of 50 mg L^−1^ ion contents. Typical CD spectra, normalized to the red absorption band, of the untreated (control) and treated samples (panels **A**, **B**). Variations of the psi-type CD amplitudes, calculated as the difference of the main positive and negative bands in the red spectral region, mean values ± SE from five independent experiments on different batches **(C)**. Different letters in panel **(C)** indicate statistically significant differences (ANOVA with Bonferroni post-hoc test, P<0.05).

As shown in **Figure 5**, both Cd (A) and Cr (B) affect moderately the chiral macroorganization of the protein complexes in the thylakoid membranes of *C. variabilis* cells. It appears that all the three psi-type bands are diminished, which suggests an overall perturbation of the long-range order of the protein complexes in the membrane—with more pronounced changes in the presence of Cd (**Figure 5A**). (N.B. the diminished magnitudes cannot be accounted for by the decreased pigment contents of the cells since the spectra are normalized to the red-absorbance maxima of the samples.)

### Distinct Effects of Cd and Cr on the Ultrastructure of Thylakoid Membranes

**Figure 6:**
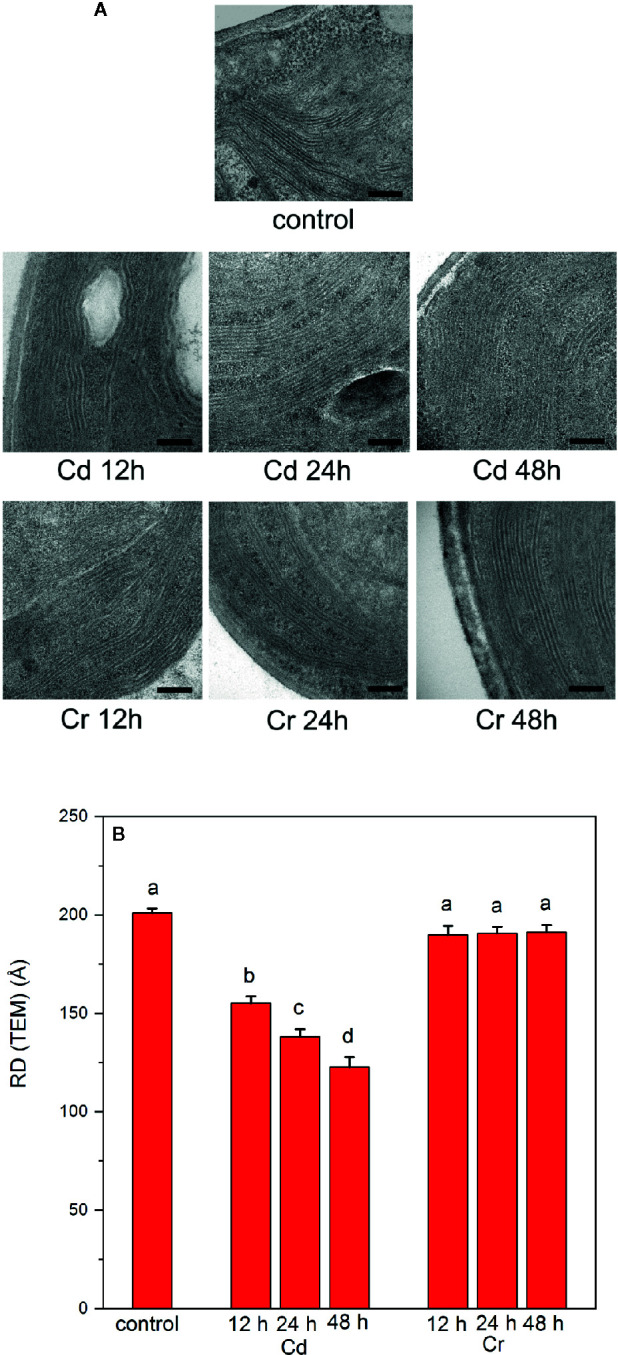
Electron microscopy images **(A)** and thylakoid-membrane repeat distances (RD(TEM)) **(B)** of untreated and trace metal ion treated *C. variabilis* cells 12, 24, and 48 h after the treatments with cadmium and chromium of 50 mg L^−1^ ion contents, compared to the untreated (control) cells. Scale bar in **(A)**, 200 nm. **(B)**: RD(TEM) values, means ± SE, obtained from 20 measurements on 10 different images. Different letters in panel **(B)** indicate statistically significant differences (ANOVA with Bonferroni post-hoc test P<0.05).

Compared to CD spectroscopy, TEM images revealed much stronger distinction between the effects of the two trace metal ions on the organization of thylakoid membranes (**Figure 6**). EM images of Cd-treated samples revealed some irregularities and membrane undulations in the presence of Cd after already 12–24 h; after 48 h the membranes became somewhat fuzzy in their appearance (**Figure 6A**). At the same time no significant alterations could be discerned in Cr-treated cells. In perfect agreement with this notion, the quantitative analysis of the lamellar repeat distances [RD(TEM)] revealed a strong shrinkage of thylakoid membranes in the presence of Cd; while Cr had no effect (**Figure 6B**). It must be noted that such a strong shrinking of the membranes cannot occur in the presence of the oxygen evolving complex, with its large volume in the lumenal aqueous space, or without strong wrinkling of the membranes. Normally, in the stacked region, RD is defined by the membrane thickness (2x40 Å), and the stroma- and lumen-side protruding proteins, the stroma exposed regions of PSII and LHCII, about 2x~20 Å), and the OEC and other smaller lumen-side LHCII and PSII protein sections (~40–45 Å) (Daum et al., 2010). Hence, the lower limit of RD is about 160–165 Å. Degradation of the PsbO protein and the disassembly of the OEC may allow the strong shrinkage, <150 Å RD(TEM) values.

The periodic organization of the multilamellar membrane system was also investigated by the non-invasive technique of small-angle neutron scattering. SANS has been shown to deliver statistically and spatially averaged information, for the entire sample volume exposed to the neutron beam, on the periodic organization of the thylakoid membranes *in vivo*, as demonstrated for cyanobacteria, algal cells and intact leaves, under physiologically relevant conditions, without fixation or staining (Nagy et al., 2011; Nagy et al., 2012; Liberton et al., 2013; Nagy et al., 2013; Nagy et al., 2014; Ünnep et al., 2014).

**Figure 7:**
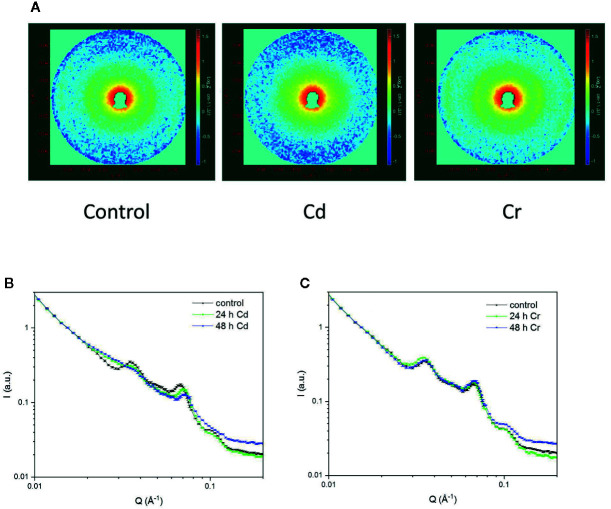
2D scattering profiles of *Chlorella variabilis* cells of untreated (left) and 48 h cadmium- (middle) and chromium-treated (right) cells **(A)**; and the 1D profiles showing the effects of cadmium **(B)** and chromium **(C)** treatments after 24 and 48 h, compared to the untreated control. The 2D images cover a momentum transfer range between 0.008 and 0.078 Å^−1^. Curves in panels **(B**, **C)** are fitted to the data points using the sum of four Gaussians and a power function.

As shown in **Figure 7A**, the 2D scattering images of the Cd- and Cr-treated samples could unequivocally be distinguished from each other. Whereas in the untreated cells and in the presence of Cr, Bragg diffraction could be clearly identified, this signature was missing in the 48 h Cd-treated sample. More quantitative analyses of the data were performed on the 1D scattering curves derived from the 2D profiles measured in low and high Q regions (**Figures 7B, C**). In particular, we fitted the scattering curves with the sum of 4 Gaussians (centered around 0.035, 0.05, 0.065, and 0.095 Å^−1^, respectively, for control samples) and a power function (cf. Nagy et al., 2013). The fitting results confirmed, that the chromium treatment has essentially no influence on the position, width or intensity of any of the Gaussians. In contrast, after 24 h cadmium treatment the intensity of the 1^st^ Gaussian—corresponding to the first order Bragg peak of the periodic thylakoid membrane structure—is reduced by c.a. 50%, and after 48 h Cd-treatment the bandwidth (FWHM) of the peak almost doubled. At the same time there is a well observable shift in the position of the 3^rd^ Gaussian peak from 0.067 ± 0.001 Å^−1^ to 0.073 ± 0.001 Å^−1^. We propose that the observed variations are a consequence of the removal of the OEC from the PSII, resulting in the reduced asymmetry of the individual thylakoid membranes (between the lumenal and interthylakoidal side). Furthermore, the removal of the OEC also may reduce the difference in size and protein composition (hence average neutron scattering length density) of the lumenal and interthylakoidal aqueous phases. All these changes result in the appearance of an extra translational symmetry in the unit cell of the thylakoid membrane from the neutron scattering point of view, and in the disappearance of the first order Bragg peak. In other terms the system of stacked flattened vesicles (half of the interthylakoidal space, membrane, lumenal space, membrane, half of the interthylakoidal space) is reduced from the scattering point of view to stacks of individual membranes separated by aqueous phases.

As discussed above the conclusions that can be deduced from TEM and SANS data are in harmony with each other. Both techniques showed that whereas Cr exerted no effect on the periodic organization of the thylakoid membranes, Cd induced a dramatic change—a very strong shrinkage and faint appearance of the membranes. It must, however, be noted that with regard to the RD values the two techniques yields somewhat different values, in the control and Cr-treated samples RD(TEM) values of ~200 Å were obtained, whereas the RD values derived from the position (Q*) of the first-order Bragg peak (RD=2π/Q*) gives 175(± 1) Å. The origin of this discrepancy is not known. The larger RD(TEM) values might be accounted for by some inherent irregularities in the membranes—undulations and wrinkles, which might increase the apparent tallness of the stacked region. Such irregularities may remain undetected by SANS. The elucidation of this question is outside the scope of the present study, and does not affect our main conclusions from TEM and SANS, i.e. the well preserved membrane ultrastructure in the presence of Cr, and its strong perturbation and loss of the characteristic features of the thylakoid membrane system in the presence of Cd, compared to the untreated cells.

### Different De-Epoxidation States of the Xanthophyll-Cycle Pigments With Cd and Cr

As part of the multilevel regulatory mechanisms developed by green algae and plants against photo-oxidative stress, the XC is of potential interest (Eskling et al., 1997). It has been shown that the toxic effects of Cr, in particular the photoinhibition of PSII in the presence of Cr(VI), depends strongly on the activity of the XC in wild type and mutant *Chlamydomonas* (Ait Ali et al., 2008). This observation prompted us to investigate the variations in the concentration of violaxanthin (Vx), antheraxanthin (Ax), and Zx, and the de-epoxidation state [DEPS=(Ax+Zx)/(Vx+Ax+Zx)] of these xanthophyll pigments in Cd- and Cr-treated *C. variabilis* cells.

During carotenogenesis XC pigments are derived from β-carotene. Zx is formed by the hydroxylation of β-carotene. Further epoxidation of Zx by Zx epoxidase (ZEP) produces Vx *via* the intermediate Ax. In the XC this reaction is reversed by Vx deepoxidase (VDE). Zx is known to play a role in the thermal dissipation of excess excitation energy (Demmig-Adams et al., 2014); in addition, it is capable of acting as antioxidant and to protect the thylakoid membranes against oxidative stress (Havaux et al., 2007). In general, the conversion of Vx to Ax and Zx and back occur rapidly, allowing rapid adjustments of the light harvesting and dissipative functions. It has, however, been shown that under photoinhibitory conditions the activity of ZEP can be dramatically down-regulated (Reinhold et al., 2008), which has been shown to be caused by the degradation of ZEP, parallel with that of D1 (Bethmann et al., 2019).

**Figure 8:**
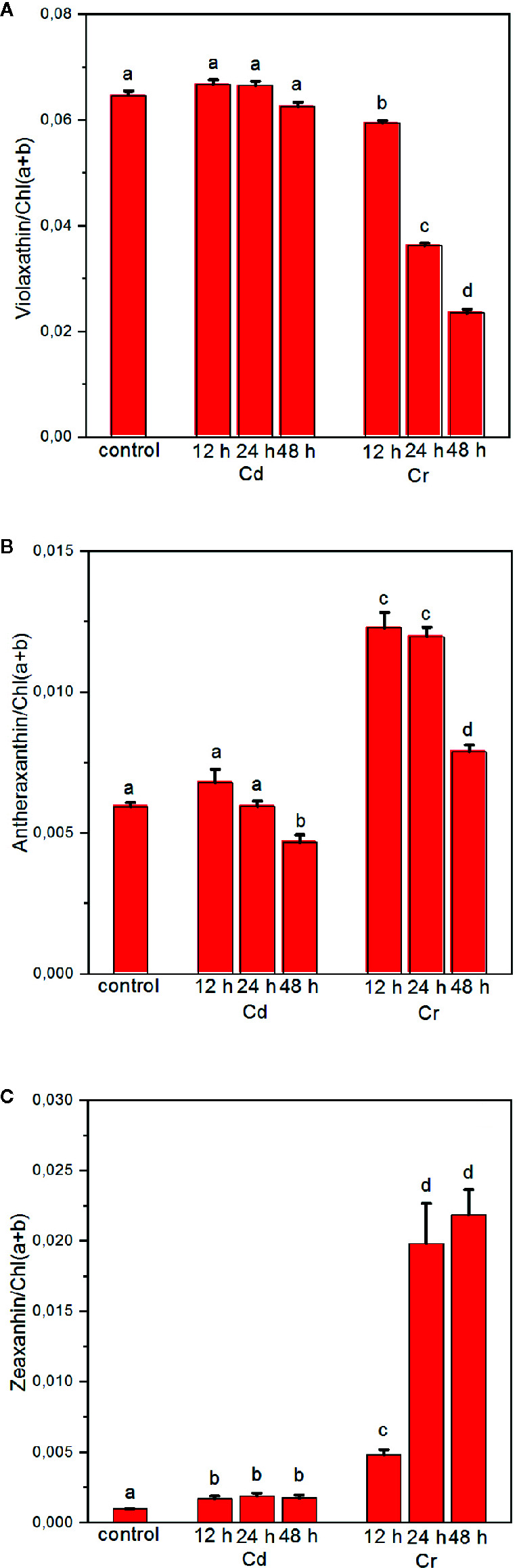
Effect of cadmium- and chromium-treatments on the composition of the xanthophyll-cycle pigments violaxanthin **(A)**, antheraxanthin **(B)** and zeaxanthin **(C)** relative to the Chl content of the cells. Mean values ± SE from five independent experiments on different batches. Different letters indicate statistically significant differences (ANOVA with Bonferroni post-hoc test, P<0.05).

Our experiments revealed that while Cd treatment exerted no significant effect on the level of XC pigments, Cr induced a marked decrease in Vx with a concomitant increase of Ax and Zx (**Figure 8**), resulting in a high DEPS. The complementary change of epoxidated and deepoxidated forms indicates that Zx and Ax accumulated at the expense of Vx due to the activity of VDE. Accumulation of Zx during the biosynthetic pathway is highly unlikely since the trace metal treatment evidently blocked all pigment synthesis and rather caused some degradation, which, on chlorophyll basis, could be seen only after 48 h of Cr treatment (cf. data in **Figure 8**). Hence, the enhanced DEPS in Cr treated cells should be attributed to the accumulation of Ax and Zx due to the activity of the XC during the cultivation of cells, and the stress-induced (partial or full) inhibition of ZEP activity. This apparently does not require the degradation of the D1 protein, which even after 48 h appears to be only marginally affected by Cr (see **Figure 3**). It is also interesting to note that in the case of Cd, the marked degradation of PSII (see **Figure 3**) does not bring about the accumulation of Zx. These data show that under stress conditions inactivation of ZEP does not necessarily require the degradation of D1 protein and degradation of this protein does not necessarily inactivate the ZEP enzyme; a question which warrants further investigations. Nevertheless, it seems very likely that the accumulated Zx—by mitigating the excitation pressure on PSII and alleviating the oxidative damage—aided the preservation PSII protein subunits and the integrity of the thylakoid membranes.

## Summary and Concluding Remarks

In this work we carried out a systematic comparison of the adverse effects of cadmium and chromium on the photosynthetic machinery of the unicellular freshwater green alga *C. variabilis*. We applied high concentrations of CdCl_2_ x 2.5 H_2_O and K_2_Cr_2_O_7_, which halted the propagation of cells but led only limited pigment bleaching, the rate of which was about the same with Cd and Cr, and its magnitude in 48 h did not exceed 40%. Although both Cd and Cr are known to exert their toxic effects *via* ROS, the inhibitory and protective mechanisms appear to be very different. The main differences were found (i) in the inhibition of PSII, which in the case of Cd could be attributed to the degradation of PsbA and PsbO proteins, whereas in the case Cr inactivation of PSII could rather be explained by an inhibition on the PSI side; (ii) with Cd, severe perturbations were detected in the thylakoid membrane system and the macro-organization of the protein complexes, which, respectively, were absent and much smaller in the presence of Cr; (iii) treatment of cells with Cr led to the accumulation of de-epoxidized xanthophylls, which could protect the thylakoid membranes against photooxidative damages; in contrast, in Cd-treated cells de-epoxidized xanthophyll molecules were found only in traces. Regarding the origin of these differences, we hypothesize, that (i) by conversion of Cr(VI) to Cr(III) in the cells, the persistence of toxic reactions is more limited than with Cd; and (ii) the sites of action might be different, and thus (iii) on molecular level, the induction of repair mechanisms and expression or silencing of genes might differ in the two cases, i.e. with Cd and Cr.

## Data Availability Statement

The original contributions presented in the study are included in the article/supplementary material, further inquiries can be directed to the corresponding authors.

## Author Contributions

ZH and OZ conceived the study. The cultures were grown and treatments applied both at the BRC and the PSI by OZ, who also carried out the CD spectroscopic and fast Chl-a fluorescence and 820 nm absorbance transient experiments. Pigment analyses and Western blot experiments were conducted by LK and OZ. RP, KS, and TP carried out the electron microscopy experiments, with the data analyzed by RP, OZ, and GG. SANS measurements were configured, performed and analyzed by GN and UG, with the participation of GG. The paper was written by ZH, GG, OZ, and LK, with all authors contributing to the writing.

## Funding

This work was supported by grants of the National Research Development and Innovation Office of Hungary (OTKA KH 124985 and K 128679), and of the Czech Science Foundation (GACR 19-13637S) to GG. GN was, in part, supported by the János Bolyai Research Scholarship of the Hungarian Academy of Sciences and by the ÚNKP-19-4 New National Excellence Program of the Ministry for Innovation and Technology. KS acknowledges the support from the Bolyai János Research Scholarship of the Hungarian Academy of Sciences. Infrastructural background for the electron microscopic evaluation was partially supported by the Ministry for National Economy of Hungary through the GINOP-2.3.3-15-2016-00001 program (RP and TP).

## Conflict of Interest

The authors declare that the research was conducted in the absence of any commercial or financial relationships that could be construed as a potential conflict of interest.
